# Robust Noise Suppression Technique for a LADAR System via Eigenvalue-Based Adaptive Filtering †

**DOI:** 10.3390/s19102311

**Published:** 2019-05-19

**Authors:** Xianzhao Xia, Rui Chen, Pinquan Wang, Yiqiang Zhao

**Affiliations:** School of Microelectronics, Tianjin University, Tianjin 300072, China; ruichen@tju.edu.cn (R.C.); pq_wang@tju.edu.cn (P.W.)

**Keywords:** LADAR, noise suppression, noise level estimation, guided image filtering

## Abstract

The laser detection and ranging system (LADAR) is widely used in various fields that require 3D measurement, detection, and modeling. In order to improve the system stability and ranging accuracy, it is necessary to obtain the complete waveform of pulses that contain target information. Due to the inevitable noise, there are distinct deviations between the actual and expected waveforms, so noise suppression is essential. To achieve the best effect, the filters’ parameters that are usually set as empirical values should be adaptively adjusted according to the different noise levels. Therefore, we propose a novel noise suppression method for the LADAR system via eigenvalue-based adaptive filtering. Firstly, an efficient noise level estimation method is developed. The distributions of the eigenvalues of the sample covariance matrix are analyzed statistically after one-dimensional echo data are transformed into matrix format. Based on the boundedness and asymptotic properties of the noise eigenvalue spectrum, an estimation method for noise variances in high dimensional settings is proposed. Secondly, based on the estimated noise level, an adaptive guided filtering algorithm is designed within the gradient domain. The optimized parameters of the guided filtering are set according to an estimated noise level. Through simulation analysis and testing experiments on echo waves, it is proven that our algorithm can suppress the noise reliably and has advantages over the existing relevant methods.

## 1. Introduction

LADAR is widely used in unmanned aerial vehicles (UAV), terrain mapping, robots, and automobile auxiliary driving. Recently, through acquiring the distance information between the receiver and the targets and through capturing the intensity signals of the echo, the LADAR system can accomplish four dimensional imaging for the targets or scenes [1]. The system emits a pulsed signal and receives the echo, which is a Gaussian pulse waveform, then a Gaussian voltage is obtained after circuit transformation [2]. Before transmitting this analog Gaussian signal to the processing system, it is necessary to use a high-speed analog-to-digital converter (ADC) to digitize the signal into discrete point data, which are related to the intensity values [1,3]. In order to obtain environment and target information, echoes need to be further analyzed to get the amplitude, pulse width, and integral intensity information [4]. All the analyzing methods are based on noise suppression, and a good noise suppression effect guarantees effective analysis [5,6].

The noise of LADAR signal is mainly Gaussian white noise, which is generally divided into internal and external noise, introduced by the avalanche photodiode (APD) in the process of photoelectric conversion [7,8]. Internal noise, which mainly consists of shot noise, thermal noise [9], generation-recombination noise, and flicker noise, comes from the electronic noise itself. The generation mechanism is the random fluctuation of voltage or current caused by the random movement of carriers [10,11,12]. External noise that comes from the external interference of the detector includes circuit noise and background light noise. In the actual photoelectric detection process, the noise of the amplification circuit is small and usually ignored [4]. Especially in the daytime, background light noise is usually much larger than the system’s natural noise. Background light noise mainly comes from natural and artificial light sources, such as the radiation from the Sun, ground, stars, atmosphere, clouds, and other sources of incidence or reflection to the detector. It also includes the interfering signal caused by the atmosphere in the transmission process [13].

For Gaussian white noise in an image, the spatial domain and transform domain filtering methods are usually used to reduce the noise. After one echo has been digitized into a one-dimensional signal, the image filtering algorithm can be used to suppress the noise of the LADAR signal. The most widely-used noise suppression methods in image processing are Gaussian algorithm [14], the bilateral filtering algorithm [15,16], and the guided filtering algorithm [17,18].

Compared with image noise, there are two special characteristics of LADAR. Firstly, multi-echo superposition will result in multiple positive and negative gradient values. The waveform is usually integrated in the time domain to analyze the material, texture, roughness, and other information of the target. Therefore, peaks and valleys need to be protected. Secondly, the light conditions, temperature, and objects around the LADAR are constantly changing [19]. As a result, it needs dynamic filter parameters that are associated with noise to adapt to the environment.

To solve the aforementioned problems, we propose a noise suppression algorithm that can accurately estimate the noise variances in two dimensions. Based on the requirement of preserving waveform boundaries, the guided filtering algorithm is used to suppress the noise of echoes, and the actual echoes are taken as the guide images. As a novel contribution, we extract the characteristic values of noise signals after separating the subspaces between the signals and the noise based on random matrix theory (RMT) [20], then we set the regularization parameters and window radius in gradient domain guided filtering. The practical usefulness of our method is illustrated by experiments.

## 2. Related Works

The echoes of LADAR are converted into digital signals after sampling and digitization by ADC. Since each echo signal is processed by an ADC of the same frequency, the number of data points of every echo is the same. Taking all data points of one echo as a one-dimensional orderly array, it can be processed by image filtering.

Image filtering is a basic module of image preprocessing, which can remove noise from images on the premise of preserving image details and textures as much as possible. Many spatial image filtering algorithms have been used for one-dimensional signal filtering [17,21,22]. The key problem of image filtering is to get a good balance between effectively removing noise and preserving detail and texture.

Image filtering generally includes the following categories: spatial filtering and transform domain filtering. Spatial image filtering calculates the neighborhood with each pixel in the image as the center and then replaces the original pixel value with the results obtained. Linear spatial filtering mainly includes box filtering, mean filtering, and Gaussian filtering, and nonlinear spatial filtering mainly includes median filtering, bilateral image filtering (BIL), and guided image filtering (GIF).

The box filter is a quick summation of the pixel values in each window within a given sliding window [23]. In the mean filter, the normalized box filter, each pixel of the output images is the average of the corresponding pixels of the input image in the window [24]. The mean filtering algorithm is simple and fast, which has a good suppression effect on the periodic interference noise. However, it destroys the detailed part of the image, while suppressing noise, thus making the image blurred. Gaussian filtering is very effective at suppressing noise that obeys a normal distribution and is widely used in image processing. It is a process of the weighted average of the whole image. The value of each pixel is obtained by the weighted average of itself and other pixel values in the neighborhood. The median filter replaces each pixel of the image with the median value of the neighborhood pixel [25]. It can protect the edge of the signal while filtering the noise, especially the pulse noise, but it will lose the texture in the uniform medium area. BIF, which has the characteristics of simple, non-iterative, and local processing, combines the spatial proximity and pixel value similarity of the image. It considers the spatial information and gray similarity at the same time and achieves the goal of preserving noise suppression [15]. The BIF is a Gaussian filter function based on the spatial distribution, so the pixels far away from the edge will not affect the pixel values on the edge much. However, because it saves high-frequency information, it has a poor effect on high-frequency noise in the color image, and pulse noise is retained as the edge. Many scholars have proposed a variety of improvement methods, such as multi-resolution bilateral filtering [26,27,28], kernel regression [29], cross bilateral filtering [30], etc. GIF is used to filter the input image through a guide image, so that the final output image is roughly similar to the initial image, but the texture part is similar to the guide image [17]. GIF can improve the computing speed while maintain the edge, so it is widely used in image enhancement, high dynamic range (HDR) compression, image matting, and haze removal [21,22].

Transform domain filtering transforms images from the spatial domain to other domains. Utilizing the properties of the transform domain, the transform coefficient is further processed to reduce the noise, and then, the image is reconstructed by inverse transformation, such as discrete cosine transform (DCT) [31], wavelet transform [32,33,34], and so on. However, due to its huge computational cost, the processed image cannot not be acquired in real time. To improve its computing speed, Stockwell proposed the discrete cosine S transform [35]. Wavelet transform was used for image noise suppression, which is a more widely-used image noise suppression method in the transform domain [36].

At present, wavelet filtering methods can be divided into the Bayesian method and non-Bayesian method. Among them, non-Bayesian methods can be divided into three types: modular maximum reconstruction filtering, wavelet threshold filtering, and the wavelet spatial correlation filtering. The mode-maximum noise suppression method mentioned by Mallat has a good noise suppression effect on both Gaussian white noise and impulse noise, but it has a large amount of computation, and the iteration is prone to instability [37]. The spatial correlation noise suppression method proposed by Xu selects the coefficients according to the strong and weak performance of the correlation between the signal and noise in each scale, and the noise filtering effect is very significant [36]. The spatial correlation noise suppression method is easy to implement, but the error is large. The wavelet threshold noise suppression method was first mentioned by Donoho [33]. The wavelet coefficient of the signal is larger than the noise, so selecting the appropriate critical threshold can remove the noise. Because threshold setting is the key to this method, many scholars devoted themselves to improving the algorithm by setting various thresholds. To overcome the visual illusion of interference caused by the critical sampling wavelet coefficient, the over-complete wavelet was used to improve this defect [38].

In combination with the characteristics of the spatial domain and the transform domain, scholars proposed the gradient domain guided image filter (GGIF) [21,39,40]. It can obtain better effects than GIF and weighted guided image filtering (WGIF) by using the gradient domain information in guiding the image as the weight.

**Figure 1:**
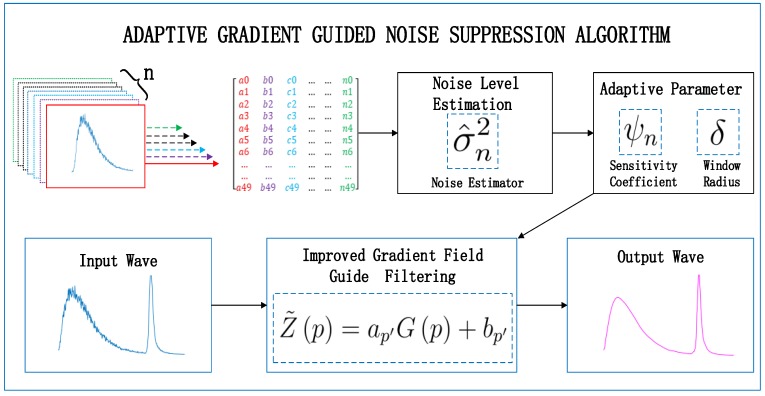
Framework of the adaptive gradient-guided noise suppression algorithm.

However, noise would change the edge part of the image, and the impact is unpredictable, so the above algorithms suffer from the fixed noise parameter. To address this, we propose an adaptive gradient-guided filtering (AGGF) algorithm, which was inspired by [21,22], based on noise estimation. Its framework is shown as Figure 1.

## 3. Adaptive Gradient-Guided Noise Suppression Technique

In [21], there were a guidance image *G* and an image to be filtered *X*, and they could be identical. Let $\Omega_{\delta_{1}}\left(p\right)$ be a square window centered at a pixel *p* of a radius $\delta_{1}$. It was assumed that the output image $\hat{Z}$ is a linear transform of the guidance image *G* in the window $\Omega_{\delta_{1}}\left(p^{\prime }\right)$:

(1) $$\hat{Z}\left(p\right)=a_{p^{\prime }}G\left(p\right)+b_{p^{\prime }},\forall p\in \Omega_{\delta_{1}}\left(p^{\prime }\right),$$

where $a_{p^{\prime }}$ and $b_{p^{\prime }}$ are two constants in the window $\Omega_{\delta_{1}}\left(p^{\prime }\right)$. Cost function $E\left(a_{p^{\prime }},b_{p^{\prime }}\right)$ with edge-aware weighting ${\hat{\Gamma }}_{G}\left(p^{\prime }\right)$ is as follows:

(2) $$E=\sum_{p\in \Omega_{\delta_{1}}\left(p^{\prime }\right)}\left[{\left(a_{p^{\prime }}G\left(p\right)+b_{p^{\prime }}-X\left(p\right)\right)}^{2}+\frac{\psi }{{\hat{\Gamma }}_{G}\left(p^{\prime }\right)}\left(a_{p^{\prime }}^{2}-\gamma_{p^{\prime }}\right)\right],$$

(3) $$\gamma_{p^{\prime }}=1-\frac{1}{1+e^{\eta \left(\chi \left(p^{\prime }\right)-\mu_{\chi ,\infty }\right)}},$$

ψ is a regularization parameter that restrains large $a_{p^{\prime }}$. $\mu_{\chi ,\infty }$ is the mean value of all $\chi \left(p\right)$. η is an adjustment coefficient that is defined as $4/\left(\mu_{\chi ,\infty }-min\left(\chi \left(p\right)\right)\right)$; it makes $a_{p^{\prime }}$ less sensitive.

(4) $${\hat{\Gamma }}_{G}\left(p^{\prime }\right)=\frac{1}{N}\sum_{p=1}^{N}\frac{\chi \left(p^{\prime }\right)+\epsilon }{\chi \left(p\right)+\epsilon },$$

${\hat{\Gamma }}_{G}\left(p^{\prime }\right)$ is an edge-aware weighting. The radius of the window is three and $\delta_{1}$ for $p^{\prime }$ and *p*, ϵ is a small positive constant. $\chi \left(p^{\prime }\right)$ is defined as $\sigma_{G,1}\left(p^{\prime }\right)\sigma_{G,\delta_{1}}\left(p^{\prime }\right)$. It is usually set to 16 in detailed manipulation applications. The weighting ${\hat{\Gamma }}_{G}\left(p^{\prime }\right)$ measures the importance of pixel $p^{\prime }$ with respect to the whole guidance image [21].

(5) $$a_{p^{\prime }}=\frac{\mu_{G⊙X,\delta_{1}}\left(p^{\prime }\right)-\mu_{G,\delta_{1}}\left(p^{\prime }\right)\mu_{X,\delta_{1}}\left(p^{\prime }\right)+\frac{\psi }{{\hat{\Gamma }}_{G}\left(p^{\prime }\right)}\gamma_{p^{\prime }}}{\sigma_{G,\delta_{1}}^{2}\left(p^{\prime }\right)+\frac{\psi }{{\hat{\Gamma }}_{G}\left(p^{\prime }\right)}},$$

(6) $$b_{p^{\prime }}=\mu_{X,\delta_{1}}\left(p^{\prime }\right)-a_{p^{\prime }}\mu_{G,\delta_{1}}\left(p^{\prime }\right).$$

where ⊙ is the element-wise product of two matrices. $\mu_{G⊙X,\delta_{1}}\left(p^{\prime }\right)$, $\mu_{G,\delta_{1}}\left(p^{\prime }\right)$, and $\mu_{X,\delta_{1}}\left(p^{\prime }\right)$ are the mean values of G⊙X, *G*, and *X* in the window $\Omega_{\delta_{1}}\left(p^{\prime }\right)$, respectively [22].

According to the influence of noise on the parameters, a new filtering algorithm is proposed.

### 3.1. Gradient-Guided Filtering

**Figure 2:**
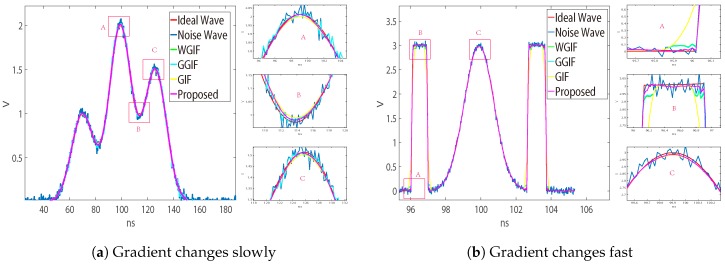
Comparison of the filtering effect. (**a**) Signal to noise ratio (SNR) = 25, $f_{p}$ = 5G, ψ = 300. δ = 7, variance = 0.01955 in weighted guided image filtering (WGIF). δ = 7, variance = 0.09954 in gradient domain guided image filter (GGIF). δ = 7, variance = 0.01987 in GIF. δ = 6–8, variance = 0.02107 in adaptive gradient-guided filtering (AGGF). (**b**) $f_{p}$ = 5G, SNR = 35, ψ = 67. δ = 5, variance = 0.01955 in WGIF. δ = 5, variance = 0.09954 in GGIF. δ = 5, variance = 0.01987 in GIF. δ = 5–7, variance = 0.02107 in AGGF.

The same as the GGIF, the key assumption of the AGGF is a local linear model between the guidance image Gand the filtering output $\tilde{Z}\left(p\right)$. It is shown that $▽\tilde{Z}\left(p\right)=a_{p^{\prime }}▽G$, and the smoothness of $\tilde{Z}\left(p\right)$ in $\Omega_{\delta }$ depends on $a_{p^{\prime }}$. The optimal values of $a_{p^{\prime }}$ and $b_{p^{\prime }}$ are computed by minimizing a new cost function, which is defined as:

(7) $$E=\sum_{p\in \Omega_{\delta }\left(p^{\prime }\right)}{\left(a_{p^{\prime }}G\left(p\right)+b_{p^{\prime }}-X\left(p\right)\right)}^{2}+\frac{\psi_{n}}{{\hat{\Gamma }}_{G}\left(p^{\prime }\right)}\left(a_{p^{\prime }}^{2}-\alpha_{n}\gamma_{p^{\prime }}\right),$$

where $\psi_{n}$ is the sensitivity coefficient of guided filtering and $\gamma_{p^{\prime }}$ is the same as Equation (3). The GGIF [21] is less sensitive to the selection of ψ, and edges could be preserved better than both the GIF and the WGIF. However, GGIF does not define where the edge is; when the overall gradient of the image does not change much, it will have a negative impact on the filtering effect, shown as Figure 2a. To avoid this, we add the parameters α to modify $\gamma_{p^{\prime }}$. When the maximum gradient is less than a threshold TH, $\alpha_{n}$ is zero, and when the maximum gradient is greater than a threshold value, $\alpha_{n}$ is one. In other words, it uses $\gamma_{p^{\prime }}$ to modify $\psi_{n}$ when the overall gradient changes a little. TH is defined as:

(8) $$TH=median\left[abs\left(\sigma_{G,\delta }^{2}\left(p^{\prime }\right)\right)+TH_{0}\right],$$

(9) $$TH_{0}=15\cdot median\left[abs\left(\sigma_{G,\delta }^{2}\left(p^{\prime }\right)\right)-median\left(\sigma_{G,\delta }^{2}\left(p^{\prime }\right)\right)\right],$$

(10) $$\delta =K_{\delta }\left[A_{\delta }{\left(f_{p}^{-1}\cdot 10^{9}\right)}^{B_{\delta }}+C_{\delta }\right]{\left(\frac{{\hat{\sigma }}_{n}^{2}}{H}\right)}^{D_{\delta }},$$

(11) $$K_{\delta }=\frac{1}{2}+\frac{1}{2}\left(Grad_{max}-Grad_{min}\right)\left(Grad_{p}-Grad_{min}\right),$$

where δ is the window radius, which is defined as Equation (10). H is the peak value of the echo signal. $A_{\delta }$, $B_{\delta }$, and $C_{\delta }$ are coefficients, which are 2.367, −0.82, and 0.286, respectively. ${\hat{\sigma }}_{n}^{2}$ is the noise level parameter that will be introduced in detail later. $K_{\delta }$ is the normalized coefficients that can adjust δ as the pixel gradient changes. $Grad_{max}$, $Grad_{min}$, and $Grad_{max}$ are the maximum gradient value of all pixels, the minimum gradient value, and the gradient value of pixel p in the guided image, respectively. In addition, due to the box filter in [17], the complexity of ${\tilde{\Gamma }}_{G}\left(p^{\prime }\right)$ is *O(N)* for an image with *N* pixels.

$\psi_{n}$ defined as Equation (12) can be adjusted according to the noise level to obtain a better filtering effect.

(12) $$\psi_{n}=A_{\psi_{n}}{\hat{\sigma }}_{n}^{2}+B_{\psi_{n}}{\hat{\sigma }}_{n}+C_{\psi_{n}},$$

where $A_{\psi_{n}}$, $B_{\psi_{n}}$, and $C_{\psi_{n}}$ are fitting coefficients, which are 5.5, 11, and 66, respectively.

(13) $$a_{p^{\prime }}=\frac{\mu_{G⊙X,\delta }\left(p^{\prime }\right)-\mu_{G,\delta }\left(p^{\prime }\right)\mu_{X,\delta }\left(p^{\prime }\right)+\frac{\psi_{n}}{{\hat{\Gamma }}_{G}\left(p^{\prime }\right)}\alpha \gamma_{p^{\prime }}}{\sigma_{G,\delta }^{2}\left(p^{\prime }\right)+\frac{\psi_{n}}{{\hat{\Gamma }}_{G}\left(p^{\prime }\right)}},$$

(14) $$b_{p^{\prime }}=\mu_{X,\delta }\left(p^{\prime }\right)-a_{p^{\prime }}\mu_{G,\delta }\left(p^{\prime }\right),$$

The final value of $\tilde{Z}\left(p\right)$ is given as follows:

(15) $$\tilde{Z}\left(p\right)={\bar{a}}_{p^{\prime }}G\left(p\right)+{\bar{b}}_{p^{\prime }},\forall p\in \Omega_{\delta }\left(p^{\prime }\right),$$

(16) $$\left\{\begin{array}{c} {\bar{a}}_{p^{\prime }}=\frac{1}{\left|\Omega_{\delta }\left(p\right)\right|}\sum_{p^{\prime }\in \Omega_{\delta }\left(p\right)}a_{p^{\prime }}\\ {\bar{b}}_{p^{\prime }}=\frac{1}{\left|\Omega_{\delta }\left(p\right)\right|}\sum_{p^{\prime }\in \Omega_{\delta }\left(p\right)}b_{p^{\prime }} \end{array}\right.$$

where ${\bar{a}}_{p^{\prime }}$ and ${\bar{b}}_{p^{\prime }}$ are the mean values of $a_{p^{\prime }}$ and $b_{p^{\prime }}$ in the window. $\left|\Omega_{\delta }\left(p\right)\right|$ is the cardinality of $\left|\Omega_{\delta }\left(p^{\prime }\right)\right|$.

When the pixel $p^{\prime }$ is at an edge, the value of ${\tilde{\Gamma }}_{G}\left(p^{\prime }\right)$ is usually much larger than one, which means edges can be preserved. In addition, the complexity of the AGGF is O(N) for an image with N pixels, which is the same as that of the GIF [17].

In order to illustrate the influence of threshold TH on the algorithm, we designed a comparison scheme. The maximum gradient in Figure 2a is less than the threshold; $\gamma_{p^{\prime }}$ is zero. At this time, the filtering effect is similar to WGIF, but it is not exactly the same. In GGIF, $\gamma_{p^{\prime }}$ is one, which causes excessive correction of $a_{p^{\prime }}$, and the filtering effect is very different.

In Figure 2b, the maximum gradient is larger than the threshold. the filtering effect is similar to GGIF, when the window is not adjusted adaptively. It should be noted that in the LADAR system, echo is expected by Gaussian noise, which rarely exceeds the threshold, so $\gamma_{p^{\prime }}$ needs to be corrected.

### 3.2. Noise Level Estimation via Eigenvalue Analysis

In this section, we analyze the asymptotical distribution of the ratio of extreme eigenvalues of a sample covariance matrix based on the limiting RTMlaw, then the noise level is estimated. To derive the relationship between the eigenvalues and noise level, we first construct the sample covariance matrix Σ.

Each element $d_{i}$ in the matrix Σ defined as Equation (17) is a data point obtained by sampling the echo. *N* is the number of columns in the matrix; *S* is the number of elements in each column. All data points of one echo is a column. *N* echos make the matrix Σ, and dimension of the matrix Σ is *S*. This matrix can be equivalent to an image to be processed, each data point being a pixel of the image. *N* is greater than *S*. The pulse width of the commonly-used pulsed laser is 5 ns, and the effective wavelength of an echo can be approximately 10 ns. The sampling frequency of the commonly-used ADC is 2 GHz, and an effective echo can obtain 20 sampling points. Therefore, in this work, *N* is at least 20.

(17) $$\Sigma =\frac{1}{S}\sum_{i=1}^{S}\left(d_{i}\cdot d_{i}^{T}\right)$$

According to the symmetric property, this matrix is decomposed into the product of three matrices: an orthogonal matrix *U*, matrix Σ, and a transpose matrix $U^{T}$, which can be selected by satisfying $U^{T}U=I$. Here, this transform process is written as:

(18) $$U^{T}\sum U=diag\left(\lambda_{1},\cdots ,\lambda_{m},\lambda_{m+1},\cdots ,\lambda_{S}\right)$$

Given $\lambda_{1}\geq \lambda_{2}\geq \cdots \geq \lambda_{S}$, we exploit the sequence of eigenvalues to enable the separation of signals from noise. To be more specific, we divide the eigenvalues into two sets $S_{0}=S_{1}\cup S_{2}$ by finding the appropriate bound in a spiked population model [41]. The echo signal lies in low-dimensional subspace, and thus, the leading eigenvalues in set $S_{1}={\left\{\lambda_{i}\right\}}_{i=1}^{m}$ are mainly contributed by it. The redundant-dimension subspace $S_{2}={\left\{\lambda_{i}\right\}}_{i=m+1}^{S}$ is dominated by the noise. Because the signals contribute very little to this latter portion, we take all the eigenvalues of $S_{2}$ into consideration to estimate the noise variance while eliminating the influence of the echo signal. Moreover, the random variables ${\left\{\lambda_{i}\right\}}_{i=m+1}^{S}$ can be considered as the eigenvalues of the pure noise covariance matrix.

Suppose the sample matrix Σ has the form $\left(N-m\right)\Sigma =HH^{T}$, where the sample entries of *H* are independently generated from the distribution $N\left(0,{\hat{\sigma }}_{n}^{2}\right)$. Then, the real matrix $M=HH^{T}$ follows a standard Wishart distribution [42]. In the high dimensional situation: $N/S\to \gamma \in \left[0,\infty \right)$ as S,N→∞, the Tracy–Widom law gives the limiting distribution of the largest eigenvalue of the large random matrix *M* [43]. Then, we have the following asymptotic expression:

(19) $$Pr\left\{\frac{\lambda_{m+1}/{\hat{\sigma }}_{n}^{2}-\mu }{\xi }⩽Z\right\}\to F_{TW1}\left(Z\right)$$

where $F_{TW1}\left(Z\right)$ indicates the cumulative distribution function with respect to the Tracy–Widom random variable. In order to improve both the approximation accuracy and convergence rate, even only with a few signal samples, we need to choose the suitable centering and scaling parameters μ,ξ [44]. By the comparison between different values, such parameters are defined as:

(20) $$\left\{\begin{array}{c} \mu =\frac{1}{N-m}{\left(\sqrt{S-\frac{1}{2}}+\sqrt{N-\frac{1}{2}}\right)}^{2}\\ \xi =\frac{1}{N-m}\left(\sqrt{S-\frac{1}{2}}+\sqrt{N-\frac{1}{2}}\right)\cdot {\left(\frac{1}{\sqrt{S-\frac{1}{2}}}+\frac{1}{\sqrt{N-\frac{1}{2}}}\right)}^{\frac{1}{3}} \end{array}\right.$$

The empirical distribution of the eigenvalues of the large sample matrix converges almost surely to the Marchenko–Pastur distribution on a finite support. Based on the generalized result in [45], when N→∞ and $\gamma \in \left[0,\infty \right)$, with probability one, we derive the limiting value of the smallest eigenvalue as:

(21) $$\lambda_{min}/\sigma_{n}^{2}\to {\left(1-\sqrt{\gamma }\right)}^{2}$$

According to the asymptotic distributions described in Equations (19) and (21), we further quantify the distribution of the ratio of the maximum eigenvalue to the minimum eigenvalue in order to detect the noise eigenvalues. Let $T_{n}$ be a detection threshold, then we find $T_{n}$ by the following expression:

(22) $$\begin{array}{c} Pr\left\{\frac{\lambda_{m+1}}{\lambda_{min}}⩽T_{n}\right\}\\ =Pr\left\{\frac{\lambda_{m+1}}{{\hat{\sigma }}_{n}^{2}}⩽T_{n}\cdot \frac{\lambda_{min}}{{\hat{\sigma }}_{n}^{2}}\right\}\\ \approx Pr\left\{\frac{\lambda_{m+1}}{{\hat{\sigma }}_{n}^{2}}⩽T_{n}\cdot {\left(1-\sqrt{N/S}\right)}^{2}\right\}\\ =Pr\left\{\frac{\lambda_{m+1}/{\hat{\sigma }}_{n}^{2}-\mu }{\xi }⩽\frac{T_{n}\cdot {\left(1-\sqrt{N/S}\right)}^{2}-\mu }{\xi }\right\}\\ \approx F_{TW1}\left\{\frac{T_{n}\cdot {\left(1-\sqrt{N/S}\right)}^{2}-\mu }{\xi }\right\} \end{array}$$

Note that there is no closed-form expression for the function $F_{TW1}$. Fortunately, the values of $F_{TW1}$ and the inverse $F_{TW1}^{-1}$ can be numerically computed at certain percentile points [41]. For a required detection probability $\alpha_{1}$, this leads to:

(23) $$\frac{T_{n}\cdot {\left(1-\sqrt{N/S}\right)}^{2}-\mu }{\xi }=F_{TW1}^{-1}\left(\alpha_{1}\right)$$

Plugging the definitions of μ and ξ into Equation (23), we finally obtain the threshold.

(24) $$\begin{array}{c} T_{n}=\frac{1}{N-m}\cdot \frac{S{\left(\sqrt{S-1/2}+\sqrt{N-1/2}\right)}^{2}}{{\left(\sqrt{S}-\sqrt{N}\right)}^{2}}\cdot \\ \left(\frac{{\left(\sqrt{S-1/2}+\sqrt{N-1/2}\right)}^{-2/3}}{{\left(S-1/2\right)}^{1/6}{\left(N-1/2\right)}^{1/6}}\cdot F_{TW1}^{-1}\left(\alpha 1\right)+1\right) \end{array}$$

When the detection threshold $T_{n}$ is known in the given probability, it means that an asymptotic upper bound can also be obtained for determining the noise eigenvalues of the matrix Σ. In general, the noise eigenvalues in the set $S_{2}$ surround the true noise variance as it follows the Gaussian distribution. The estimated largest eigenvalue $\lambda_{m+1}$ should be no less than ${\hat{\sigma }}_{n}^{2}$. The known smallest eigenvalue $\lambda_{S}$ is no more than ${\hat{\sigma }}_{n}^{2}$ by the theoretical analysis [46]. The location and value of $\lambda_{m+1}$ in *S* are obtained by checking the bound $\lambda_{m+1}\leq T_{n}\cdot \lambda_{S}$ with high probability $\alpha_{1}$.

Without requiring the knowledge of the signal, the threshold $T_{n}$ can provide good detection performance for finite S,N, even when the ratio N/S is not too large. Based on this result, more accurate estimation can be obtained by averaging all elements in $S_{2}$. Hence, the maximum likelihood estimator of ${\hat{\sigma }}_{n}^{2}$ is:

(25) $${\hat{\sigma }}_{n}^{2}=\frac{1}{S-m}\sum_{j=m+1}^{S}\lambda_{j}$$

Based on the above content, we propose a noise suppression algorithm as Algorithm 1.

**Algorithm 1** Noise suppression algorithm.
1:**procedure** (Input: $X,N,S,\alpha_{1},\alpha_{2},f_{p}$)  ▹*X* is input waveform; *N* is the number of echoes; *S* is thenumber of data points in each echo; $\alpha_{1}$, $\alpha_{2}$ are probability levels; $f_{p}$ is frequency of the ADC.2:    $\Sigma \leftarrow \left\{X_{1},X_{2},\cdots ,X_{N}\right\}$                          ▹Σ is data matrix3:    $\left\{\lambda_{1},\lambda_{2},\cdots ,\lambda_{S}\right\}\leftarrow \Sigma$4:    ${\hat{\sigma }}_{n}^{2}\leftarrow \left\{\lambda_{S},\cdots ,\lambda_{m+1}\right\}$                 ▹${\hat{\sigma }}_{n}^{2}$ is the maximum likelihood estimator5:    $T_{n}\leftarrow \left\{\lambda_{min},\lambda_{m+1}\right\}$                   ▹$T_{n}$ is the low-dimensional threshold6:    $\psi_{n}\leftarrow {\hat{\sigma }}_{n}^{2}$                               ▹$\psi_{n}$ is the sensitivity7:    $\delta \leftarrow f_{p}$                               ▹δ is the window radius8:    $\left\{a_{p^{\prime }},b_{p^{\prime }}\right\}\leftarrow \left\{\delta ,\psi_{n}\right\}$                 ▹$a_{p^{\prime }},b_{p^{\prime }}$ are the filter kernel parameters9:    ${\tilde{Z}}_{N+1}\left(p\right)\leftarrow \left\{a_{p^{\prime }},b_{p^{\prime },X_{N+1}}\right\}$                 ▹${\tilde{Z}}_{N+1}\left(p\right)$ is the output image10:    **return** ${\tilde{Z}}_{N+1}\left(p\right)$11:
**end procedure**



## 4. Experimental Results

**Figure 3:**
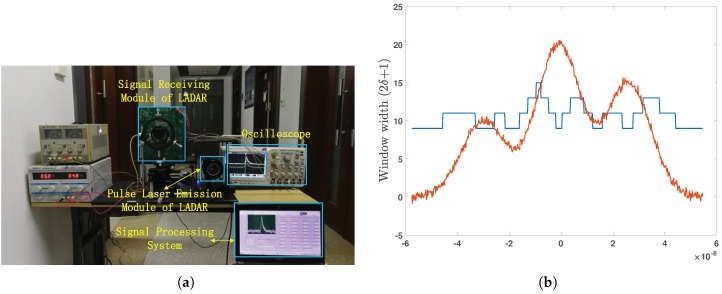
(**a**) LADAR system; (**b**) window width (2δ+1) changing with gradient.

We studied the influence of the noise on filter parameters through a large number of data simulations. Experiments on land and simulation under water were carried out to verify the effectiveness of the algorithm. As shown in Figure 3a, all measured data in this paper were obtained by the LADAR system, which used an 8 × 8 array APDas the detector and a 1064-nm pulsed laser as the detection source to realize target distance detection based on the time-of-flight method. The system uses a multi-channel preamplifier circuit based on the variable gain amplifier (VGA) and implements full waveform sampling analysis based on a 2-GHz sampling rate ADC.

### 4.1. Evaluation

**Figure 4:**
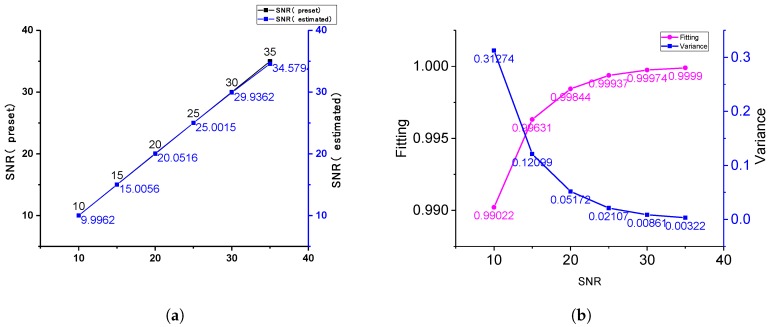
Algorithm simulation results. (**a**) Noise estimated result; (**b**) noise suppression results under different SNRs.

As shown in Figure 4a, the maximum deviation of estimation was 1.2%, while the SNR was in the range of 0–35. When the SNR is low, the eigenvalue of noise in signal matrix is large, which can be extracted more accurately, and the estimation is better. In Figure 4b, it can be seen that a good noise suppression effect can be achieved, and the fittings were more than 99% at different SNRs.

### 4.2. Analysis of the Relationship Between the Noise and Parameters

Table 1 shows the comparison of the filtering effects of these algorithms with different sensitivities at different SNR. The optimal sensitivity of WGIF, GGIF, AGGF, and GIF were approximately equal at the same SNR, and this can be obtained by comparing Equations (2) and (7). In other words, all of them optimized the edge filtering effect by weighting the $\psi_{n}$.

**Figure 5:**
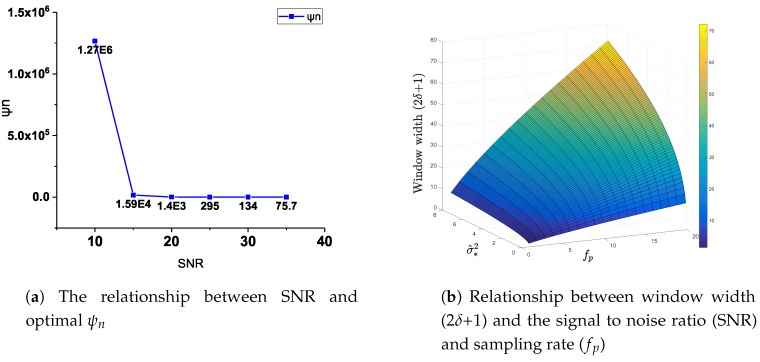
The relationship between $\psi_{n}$, window width (2δ+1), $f_{p}$, and SNR. (**a**) The relationship between SNR, optimal $\psi_{n}$ in AGGF. (**b**) Relationship between window width (2δ+1), noise level (${\hat{\sigma }}_{*}^{2}$), and sampling rate ($f_{p}$) in AGGF.

Figure 5a shows the relationship between the optimal $\psi_{n}$ and SNR of the AGGF algorithm. Due to $\psi_{n}$ changing with SNR, the proposed AGGF algorithm can achieve a good noise suppression effect at different noise levels. It can be obtained that the larger the SNR, the smaller the optimal sensitivity. Figure 5b shows the relationship between $\psi_{n}$, window width (2δ+1), $f_{p}$, and SNR.

For the LADAR waveform, the sampling rate of the ADC determines the number of data points of one echo, which is directly related to the window radius. The higher the sampling rate, the larger the window width should be.

At the same time, in order to achieve better filtering, the noise level should also be considered when setting the window width.

The proposed algorithm can adjust filter parameters according to different noise levels. To make the comparison fairer, all window radii were optimized by Equation (10). It can be observed that the effect of indoor experiment was not as good as that of the underwater experiment, and the detailed reasons will be analyzed later.

Although the filtering effect can be reflected by the two parameters of mean variance and fitting degree, they cannot fully reflect the filtering effect. For example, at the sampling rate of 2.5 GHz, WGIF and GIF had the best parameters when δ was three and five, respectively.

The unsmoothed waveform needs to be processed again to obtain the centroid position, which was used for the accurate calculation of the target distance. In contrast, the proposed adaptive window width achieved a good filtering effect at different sampling rates and SNR. Specifically, GGIF can enhance the boundary effect with parameter $\gamma_{p}^{\prime }$, but in the case of Gaussian echo, where the boundary is not obvious or the overall gradient change is not dramatic, excessive boundary judgment will occur.

In order to obtain a better filtering effect, we make the window radius larger in the part with a high slope and smaller in the part with a low slope, as Figure 3b shows.

### 4.3. Analysis of Measurement and Simulation Data

**Figure 6:**
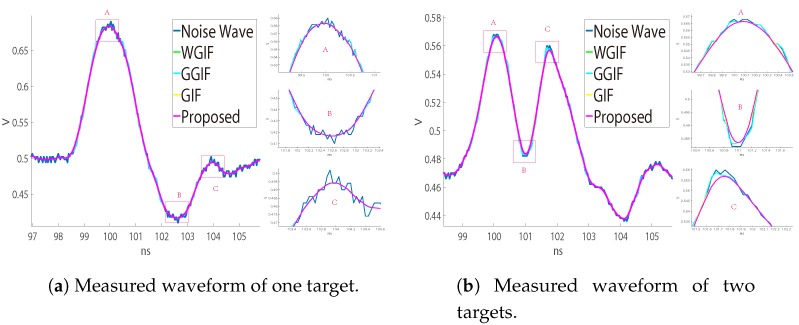
Measured waveform noise suppression effect. (**a**) Comparison of noise suppression effect of one target. (**b**) Comparison of noise suppression effect of two targets.

**Figure 7:**
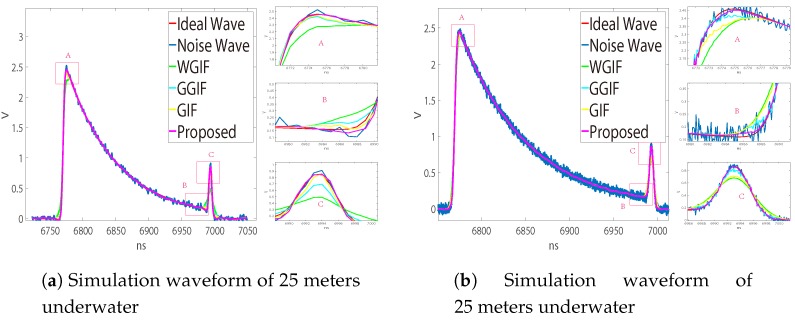
Measured waveform noise suppression effect. Comparison of noise suppression effect. (**a**) Filtering effect at $f_{p}$ = 1 GHz, SNR = 30. δ = 4 in WGIF, GGIF, and GIF, δ = 3–5 in AGGF (proposed). (**b**) Filtering effect at $f_{p}$ = 7.5 GHz, SNR = 30. δ = 11 in WGIF, GGIF, and GIF, δ = 9–13 in AGGF (proposed).

**Figure 8:**
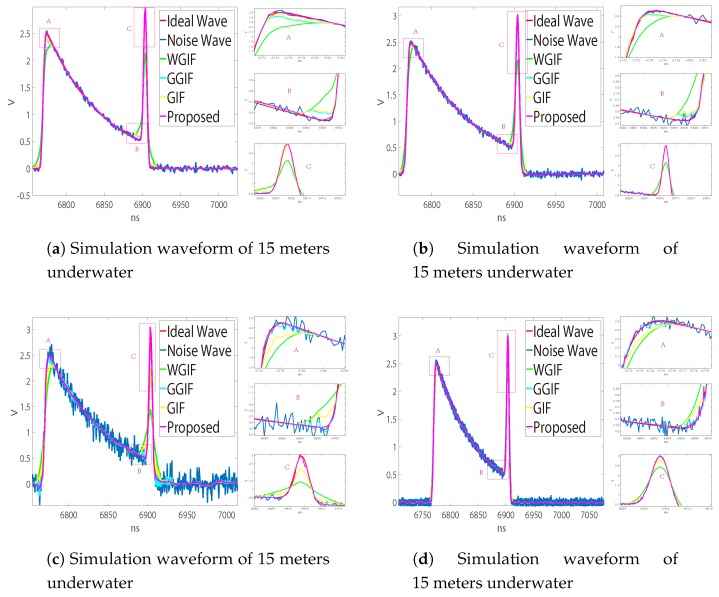
Measured waveform noise suppression effect. (**a**) Filtering effect at $f_{p}$ = 1 GHz, SNR = 30. δ = 4 in WGIF, GGIF, and GIF, δ = 3–5 in AGGF (proposed). (**b**) Filtering effect at $f_{p}$ = 2 GHz, SNR = 30. δ = 6 in WGIF, GGIF, and GIF, δ = 4–8 in AGGF (proposed). (**c**) Filtering effect at $f_{p}$ = 2 GHz, SNR = 20. δ = 10 in WGIF, GGIF, and GIF, δ = 8–12 in AGGF (proposed). (**d**) Filtering effect at $f_{p}$ = 7.5 GHz, SNR = 30. δ = 11 in WGIF, GGIF, and GIF, δ = 9–13 in AGGF (proposed).

Figure 6a,b is the waveforms measured by the LADAR system on land, in which (a) is about one object and (b) is the waveform measured by two objects 30 cm apart. Since there was no obvious gradient change, the noise suppression effect of GGIF was poor, and the effect of the other algorithms was similar. Figure 7 and Figure 8 show the underwater waveforms simulated at 15 m and 25 m, respectively. The two echoes before and after were the surface signal and underwater signal, separately. Because the light beam reflected continuously in the water, the first echo had trailing.

It can be seen in Section 3 that WGIF and GIF had a good filtering effect on waveforms with a small gradient, and they can maintain edge features well at the same time, but the edge protection effect was poor in waveforms with a large gradient.

GGIF enhanced the edge detection based on WGIF and GIF, and it was more sensitive to the edge. When the gradient was small, there would be excessive protection of the edge, which can be seen in Figure 2. We hoped that the algorithm can better distinguish the two cases, so we increased the threshold to judge the gradient change. In the case of a large gradient, the proposed algorithm approximated the GGIF, as shown in the Figure 7 and Figure 8. In the case of small gradient, the proposed algorithm approximated the WGIF and GIF, as shown in the Figure 6.

In the seabed detection, due to the change of the seabed height covered by the divergence of the beam and the scattering effect of sea water, the echo gradient will change to different degrees. When the seawater is shallower, the laser energy absorbed by seawater is less, and the echo power is larger, which leads to a big gradient of echoes. The gradient of the echo signal varies with the fluctuation of the seabed. Therefore, the proposed algorithm can achieve better results in different conditions for seabed echoes. In the laboratory experiment, considering that the echo power is too large to produce a saturated signal, it is impossible to carry out effective analysis. Therefore, in the indoor test, it is necessary to add an attenuation lens to the laser transmitting lens to reduce the laser power and at the same time reduce the gain of the signal processing circuit, which will reduce the echo gradient. Therefore, in the comparison of the indoor signal filtering effect, the proposed algorithm has no obvious advantages.

As shown in Figure 8, when the underwater echo is very strong, the advantage of the proposed algorithm is more obvious, as a result of adjusting parameters reasonably according to the noise level and gradient. As shown in Figure 7, when the underwater echo is very small, the AGGF noise suppression effect is still better. Therefore, this proposed method can be better applied to noise suppression of the LADAR waveform.

**Figure 9:**
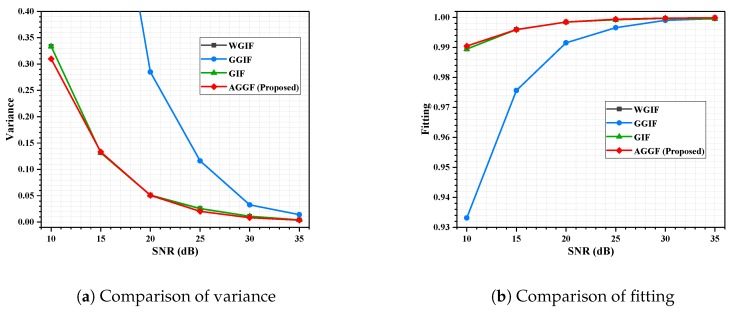
Indoor experiment, $f_{p}$ = 5 GHz; influence of SNR on filtering effect.

**Figure 10:**
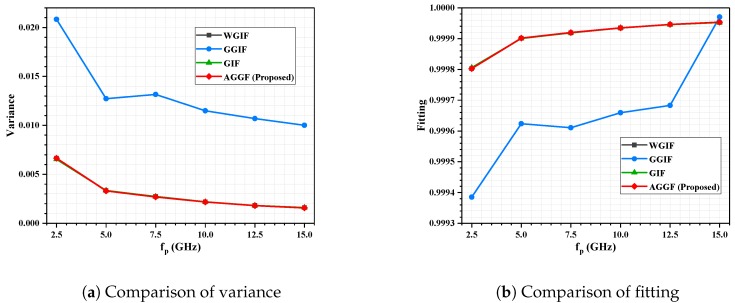
Indoor experiment, SNR = 30; influence of $f_{p}$ on filtering effect.

**Figure 11:**
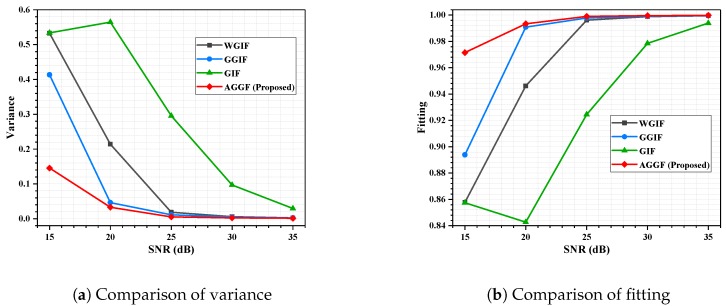
Seabed experiment, $f_{p}$ = 2 GHz; influence of SNR on filtering effect.

**Figure 12:**
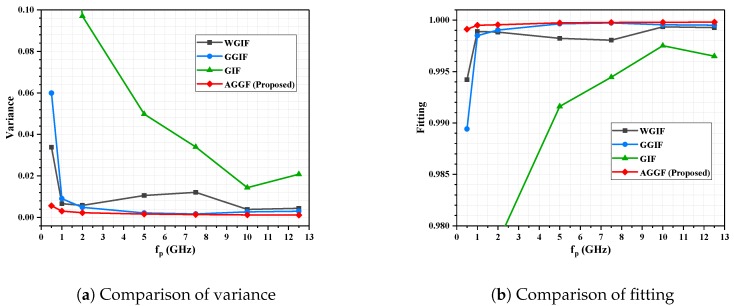
Seabed experiment, SNR = 30; influence of $f_{p}$ on filtering effect.

Detailed simulation results are shown in Figure 9, Figure 10, Figure 11 and Figure 12. It can be seen that the algorithm proposed is more advantageous in the application of a large gradient, such as underwater.

## 5. Conclusions

To infer the noise level from a signal matrix, the eigen-spaces of the signal and noise are transformed and separated well by determining the eigen-spectrum interval. In addition, the developed estimator can effectively eliminate the estimation bias of a noise variance in a high-dimensional context. The experimental results have demonstrated that the proposed noise suppression technique for the LADAR system via eigenvalue-based adaptive filtering can adjust parameters according to the noise level, and it outperforms the relevant existing methods over a wide range of noise level conditions for LADAR system.

## Figures and Tables

**Table 1:** Influence of SNR on $\psi_{n}$ for different filtering algorithms, $f_{p}$ = 5G. SNR = 35, $\psi_{n}$ = 69, δ = 4 in WGIF, GGIF and GIF, δ = 3–5 in AGGF; SNR = 30, $\psi_{n}$ = 134, δ = 6 in WGIF, GGIF and GIF, δ = 3–7 in AGGF; SNR = 25, $\psi_{n}$ = 295, δ = 7 in WGIF, GGIF and GIF, δ = 4–8 in AGGF; SNR = 20, $\psi_{n}$ = 1400, δ = 8 in WGIF, GGIF and GIF, δ = 6–10 in AGGF; SNR = 15, $\psi_{n}$ = 5800, δ = 9 in WGIF, GGIF and GIF, δ = 8–12 in AGGF; SNR = 10, $\psi_{n}$ = 1,270,000, δ = 20 in WGIF, GGIF and GIF, δ = 12–16 in AGGF. The best results have been bolded.

SNR	$\psi_{n}$	WGIG		GGIF		GIF		AGGF (Proposed)	
		Fitting	Variance	Fitting	Variance	Fitting	Variance	Fitting	Variance
10	5000	0.9896289	0.3320057	0.9294678	2.4700421	0.9899139	0.3224299	**0.9899558**	**0.3200555**
	150,000	0.9900612	0.3242041	0.9335110	2.3769678	0.9900623	0.3241507	**0.9904550**	**0.3120494**
	1.27 × 10^6^	0.9899291	0.3223799	0.9323638	2.3626738	0.9899292	0.3223750	**0.9901822**	**0.3134338**
15	1500	0.9959852	0.1317777	0.9752516	0.8424215	0.9961218	0.1271757	**0.9962622**	**0.1220978**
	5000	0.9964259	0.1172305	0.9761854	0.8110346	0.9964482	0.1164678	**0.9965838**	**0.1120026**
	15,000	0.9962935	0.1218438	0.9757185	0.8274727	0.9962985	0.1216689	**0.9963667**	**0.1190786**
20	100	0.9979770	0.0674745	0.9907810	0.3124294	0.9983977	0.0532925	**0.9984316**	**0.0520335**
	1500	0.9985062	0.0498123	0.9913531	0.2929273	**0.9985161**	**0.0494753**	0.9985083	0.0496394
	5000	0.9984113	0.0526354	0.9913088	0.2925424	0.9984137	0.0525548	**0.9984236**	**0.0520893**
25	10	0.9986424	0.0455014	0.9966124	0.1142694	0.9993092	0.0230704	**0.9993714**	**0.0209918**
	100	0.9993676	0.0212017	0.9969995	0.1014306	**0.9994068**	**0.0198746**	0.9994056	0.0199305
	1500	0.9993093	0.0230395	0.9969920	0.1014459	0.9993100	0.0230151	**0.9993253**	**0.0225601**
30	10	0.9996188	0.0127846	0.9989465	0.0355396	0.9997132	0.0095981	**0.9997371**	**0.0088228**
	100	0.9997270	0.0091296	0.9989876	0.0341289	0.9997288	0.0090679	**0.9997469**	**0.0084929**
	1500	0.9997136	0.0095818	0.9990168	0.0331666	0.9997134	0.0095874	**0.9997366**	**0.0088431**
35	10	0.9998705	0.0043549	0.9996337	0.0123681	0.9998903	0.0036829	**0.9999041**	**0.0032282**
	100	0.9998890	0.0037272	0.9996338	0.0123681	0.9998890	0.0037263	**0.9999034**	**0.0032540**
	1500	0.9998865	0.0038077	0.9996364	0.0122755	0.9998865	0.0038097	**0.9999019**	**0.0033016**
